# Differences in motor learning-related structural plasticity of layer 2/3 parvalbumin-positive interneurons of the young and aged motor cortex

**DOI:** 10.1007/s11357-024-01350-6

**Published:** 2024-09-30

**Authors:** Andrew M. Davidson, Hernán Mejía-Gómez, Bryn M. Wooten, Sharai Marqués, Michael Jacobowitz, Irene F. Ugidos, Ricardo Mostany

**Affiliations:** 1 Department of Cell and Molecular Biology, Tulane University, New Orleans, LA USA; 2 Department of Pharmacology, Tulane University School of Medicine, New Orleans, LA USA; 3 Neuroscience Program, Brain Institute, Tulane University, New Orleans, LA USA; 4 Brain Institute, Tulane University, New Orleans, LA USA

**Keywords:** Aging, Axon, Cortex, Interneuron, Motor learning, Structural plasticity

## Abstract

Changes to neuronal connectivity are believed to be a key factor in cognitive impairments associated with normal aging. Because of its effect on activities of daily living, deficient motor control is a critical type of cognitive decline to understand. Diminished inhibitory networks in the cortex are implicated in such motor control deficits, pointing to the connectivity of inhibitory cortical interneurons as an important area for study. Here, we used chronic two-photon microscopy to track the structural plasticity of *en passant* boutons (EPBs) of parvalbumin-positive interneurons in the mouse motor cortex in the first longitudinal, in vivo study of inhibitory interneuron synapses in the context of aging. Young (3–5 months) and aged (23–28 months) mice underwent training on the accelerating rotarod to evoke motor learning-induced structural plasticity. Our analysis reveals that, in comparison with axons from young mice, those from aged mice have fewer EPBs at baseline that also tend to be larger in size. Aged axons also express learning-related structural plasticity—like new bouton stabilization and bouton enlargement—that is less persistent than that of young axons. This study reveals striking baseline differences in young and aged axon morphology as well as differences in the deployment of learning-related structural plasticity across axons.

## Introduction

A long-standing question related to the basic biology of aging is to what degree changes in neuronal connectivity are involved in normal aging-related impairments. Many studies have reported diverse changes to the structural elements of connectivity in the aged brain, including changes to dendritic branches and spines [1–7] as well as axonal arbors and boutons [1, 2, 8], implying a connection with cognitive impairment. Excitatory neurons remain the primary focus of these efforts (with one exception [5]), but as inhibitory interneurons continue to demonstrate their critical role in brain function, our need to understand the effects of aging on this important neuronal population becomes more critical.

Of the wide range of aging-related impairments, motor skill deficits are among the most critical since motor function quality is a significant indicator of healthy aging [9]. In particular, motor learning is of interest because advanced age not only requires people to learn new motor skills but often requires them to learn to do known skills in new ways [10] and to relearn previously known skills, for example during recovery from an injury or stroke. Importantly, functional decline of inhibitory networks in the aged sensorimotor cortices [11, 12] is implicated in motor control deficits [13, 14]. And along these lines, compromised inhibition is also reported in the aged mouse sensorimotor cortices [15, 16]. The population of interneurons that supply this inhibition are diverse but can be broadly divided into three primary groups [17], the largest of which is the parvalbumin-positive interneurons (PV INs). The PV IN is an attractive candidate for studying the structural plasticity associated with motor learning in the context of aging for several reasons. These interneurons directly shape and are driven by cortical output [18]. During motor learning, the *en passant* boutons (EPBs) of PV IN axons are reported to increase in density [19], and during behavior, PV INs are involved in maintaining the balance between cortical excitation and inhibition [20]. This study used the accelerating rotarod as a model of motor learning since it is known to evoke plasticity in the cortex [6, 21–23]—specifically in PV INs [24]—and because it lends itself to age group comparisons, given that it does not rely on diet or water restriction or motivation to explore, all of which are influenced by age [25].

In this study, we used in vivo two-photon microscopy to track the motor learning-related structural plasticity of EPBs along the axons of PV INs in the forepaw area of the primary motor cortex (M1) in young (3–5 months) and aged (23–28 months) mice. To our knowledge, this is the first report of long-term in vivo imaging of any interneuron synapses in the context of aging. Our analysis of EPB density, dynamics, and morphology uncovered baseline differences in EPB size and density and suggests that aged PV IN axons are subject to aging-related deficits in the maintenance of learning-related structural changes. Such changes reflect the expectation of the field that synapse size is an indicator of synapse strength [26, 27], possibly via molecular mechanisms based on physical changes associated with learning-evoked increase in the space available for readily releasable vesicles [28, 29]. The field also predicts the involvement of transient systems-level mechanisms, including plasticity-promoting arousal states and increases in cortical excitability [30]. Regardless of the mechanics of the plasticity, the results presented here indicate that the plastic effects of motor learning on PV IN axons are differently incorporated in the young and aged brain.

## Methods

### Animals

The experimental animals were the progeny of a cross between a line expressing eGFP under the Thy-1 promoter (The Jackson Laboratory, 007788, Tg (Thy1-EGFP)MJrs/J) and a line expressing Cre recombinase under the endogenous Pvalb promoter (The Jackson Laboratory, 008069, B6;129P2-Pvalb^tm1(cre)Arbr^/J), resulting in Thy-1-EGFP::PV-Cre animals whose cortex is sparsely labeled by eGFP in pyramidal neurons (PNs) and the ability to label PV INs via injection of adeno-associated virus (AAV) with Cre recombinase-dependent activity. Young animals were 3–5 months old (*N* = 6), and aged animals were 23–28 months old (*N* = 6). Animals of both sexes were used in equal numbers and pooled. The reduced sample size per group hindered any subsequent subanalysis by sex of the animals. All animals were virgins and were group housed with cagemates of the same sex and age. If at any point an animal became unable to complete the rotarod task or the condition of the cranial window did not allow quality imaging, the animal and its associated data, as well as the data from the paired animal, were excluded from the analysis. Food and water were available ad libitum, and cages were kept under a 12-h light/dark cycle. The study was carried out in accordance with the recommendations of the NIH Office of Laboratory Animal Welfare’s Public Health Service Policy on Humane Care and Use of Laboratory Animals and Guide for the Care and Use of Laboratory Animals, and all procedures described were approved by the Institutional Animal Care and Use Committee of Tulane University.

### Cranial window procedure

Implantation of glass-covered cranial windows was carried out as previously described [31, 32] but with the addition of an intracortical AAV injection to label PV INs. Briefly, animals were anesthetized with isoflurane (5.0% for induction, 1.5–2.0% for maintenance), and the skull was fastened into place on a stereotaxic frame. Dexamethasone (0.2 mg/kg bw) and carprofen (5.0 mg/kg bw) were injected subcutaneously. The occasional case of cerebral edema was controlled with a single subcutaneous injection of hypertonic saline (11.7%, 390.0 mg/kg bw). Using a pneumatic drill, a 3 mm diameter circular craniotomy was performed directly above the forepaw area of the M1, centered at 0.25 mm anterior and 1.60 mm lateral to bregma [33]. All animals received 1–3 injections of a Cre recombinase-dependent AAV cocktail after the bone fragment was removed. Then a 3 mm glass coverslip was gently placed into the craniotomy and secured to the surrounding skull with cyanoacrylate-based glue. The remaining exposed skull was covered with dental acrylic. A titanium bar was embedded into the acrylic to secure the mouse onto the imaging stage during later in vivo imaging. Between 25 and 35 days passed before the first imaging session to allow for recovery.

### AAV injection

All animals received AAV injection during the cranial window procedure. Following the removal of the bone fragment during the craniotomy, a combination of two AAVs was injected intracortically at a depth of 300 µm to target Cre recombinase-expressing PV INs in L2/3. A micropipette puller (Model P-1000, Sutter Instruments) was used to prepare injecting pipettes with approximately 0.5 µm tips and 8–10 mm tapers from glass capillary tubes (#3–000-203-G/X, Drummond Scientific). Injections were controlled with a programmable nanoliter injector (Nanoject III, Drummond Scientific) that injected 5.0 nL of virus mixture at a rate of 1.0 nL per second. This sequence was repeated 4 times at each injection site with a delay interval of 30 s. Each animal received injections at 1–3 sites. The injectable mixture contained 0.5 µL of AAV1.CAG.Flex.eGFP.WPRE.bGH (Addgene #51,502-AAV1; stock concentration of 2.96E + 13 vg/ml), 2.0 µL of AAV1.CAG.Flex.tdTomato.WPRE.bGH (Addgene #51,503-AAV1; stock concentration of 4.49E + 13 vg/ml), and 47.5 µL of 0.9% sterile saline. It was prepared fresh before each procedure. After back-filling the pipette with light mineral oil, mounting it in the injector, and front-filling it with the AAV mixture, the pipette was lowered toward the exposed brain. Its position was noted at the point where it first contacted the dura. From there, the pipette was lowered 400 µm, puncturing the dura, and was then raised 100 µm. After a 2-min wait, the injection program was initiated. The injection program lasted about 140 s. After its completion and another 2-min wait, the pipette was slowly withdrawn. The exposed brain was kept under a drop of 0.9% saline and moistened Gelfoam (Pfizer) throughout the process.

### In vivo two-photon imaging

A custom-built two-photon microscope, a Ti:Sapphire laser (Chameleon Ultra II; Coherent Inc.) tuned to 910 nm, and a 40X 0.8 NA water immersion objective (Olympus) were used for in vivo imaging. Animals were anesthetized with isoflurane (5.0% for induction, 1.0–1.8% for maintenance) throughout each imaging session, which typically lasted 20–40 min. ScanImage 3.8 software [34] was used to collect images. The field of view beneath the cranial window was scanned at a depth of approximately 150–250 µm from the dura, and the sparsely labeled axons were selected for imaging. The targeted axon was confirmed as belonging to a PV IN by verifying that it expressed both eGFP and tdTomato (tdT). As expected, tdT-expressing axons were never found within L1. The tdT signal was used to verify the cell type as PV IN, and the stronger eGFP signal was used for the morphological analysis. The same fragments of axons were imaged in subsequent sessions at high magnification (512 × 512 pixels, 0.152 µm/pixel, 1.5 µm z-steps) using a coordinate plane built around landmark vasculature and the regions of interest. High magnification imaging of axons occurred at Days 1–9 and 26. All imaging sessions took place between 9:00 am and 2:00 pm.

### Analysis of axonal bouton size, density, and dynamics

High magnification images of fragments of axons were analyzed using image analysis software written in MATLAB. Image files were randomly renamed (Bulk Rename Utility, TGRMN Software) and shuffled to guarantee that analysis was completed blind to age group. For each axonal fragment, an “empty” section of axon was transversely measured at each timepoint. All visible axonal boutons were then annotated precisely through their greatest transverse length according to the following criteria: boutons must be greater in width than the image’s corresponding “empty” axon width, and boutons must be qualitatively brighter in fluorescence than the immediately surrounding axon. A total of 1214 distinct *en passant* boutons on 2.8 mm of axon of 6 young adult animals and a total of 1258 distinct *en passant* boutons on 3.28 mm of axon of 6 aged animals were followed through 11 imaging sessions over 26 days. A new persistent bouton was defined as a gained bouton that was still present at the following imaging session, 24 h later, and a transient bouton was defined as a gained bouton that was not present at the following imaging session. The survival curves were generated by fitting the survival fraction (of all boutons present at Day *n* that remain at each subsequent day) to a one-phase exponential decay curve following plateau. X_0_ was constrained at Day 2 for pre-training data, Day 4 for forward training data, and Day 7 for reverse training data. Y_0_ was constrained at 1. The survival fractions and rate constants are calculated as survival fraction = plateau + unstable fraction X ^e−t/τ^, where *t* = time in days and *τ* = time constant; rate constant = 1/τ. Since bouton density is a static measure, data from each day of imaging can be pooled to calculate averages for each stage of training. For dynamic measures, data is only pooled from days in which the metric is completely contained within the stage and not influenced by adjacent stages. For example, EPB turnover per micron for the pre-training stage only includes data from EPBs gained or lost between Day 1 and 2 as well as Day 2 and 3, but not between Day 3 and 4, since forward training occurs between these imaging sessions. For the individual bouton width subanalysis based on permanence: boutons were classified as stable if they were present throughout the entire experimental timeline, and they were classified as dynamic if they were not.

### Motor training

Animals were trained on the accelerating rotarod. A RotaRod Rotamex 5 (Columbus Instruments) or a modified Rota-Rod 2 (Med Associates, Inc.) were used for training. To maintain consistency between the instruments, the Rota-Rod 2 was modified by replacing its spindle with a Rotamex 5 spindle. Each daily session consisted of 6 trials, and the animal received a 10-min rest period (in their home cage) between each trial. Each trial began with the placement of the animal on the rod spinning at 5 rpm, and the speed increased by an additional 5 rpm on a 20 s interval. If the animal fell from the rod within the first 5 s of the trial, the trial was re-attempted after a 10-min rest period. Otherwise, the “time to fall” was recorded for each trial with a maximum trial duration of 200 s for aged animals. The maximum trial duration for young animals was determined by randomly pairing each young animal with an aged animal and capping the young animal’s training time for each trial at the aged animal’s “time to fall.” This pairing strategy was used to ensure that any structural plasticity differences observed were due to differences of age and not differences in time spent practicing the motor skill, since the average young animal can outperform the average aged animal on this task [35]. Forward training was carried out on Nights 3, 4, and 5, and reverse training was carried out on Nights 6, 7, and 8. There was a minimum of 2 h separating the conclusion of an animal’s imaging session and the beginning of its motor training session. All parameters were the same for forward and reverse training except that a plastic barrier was added during reverse training to deter the animal from turning around. All training sessions took place between 4:00 pm and 8:00 pm.

### Analysis of forepaw position during training

All trials were recorded by a webcam (Logitech C615) mounted at the front of the rotarod instrument and positioned so that the right forepaw was consistently visible. Videos were analyzed post hoc for right forepaw position using Tracker 5.0.7 (Douglas Brown, Open Source Physics). The distance from the right forepaw to the top of the rod was measured every 2 s [36]. This analysis was completed for every animal’s best performing trial on the first and last day of forward training (Nights 3 and 5) as well as the first and last day of reverse training (Nights 6 and 8).

### Overview of study design

In vivo imaging was completed in 10 sessions over nearly 1 month, during which the animals underwent motor training via the accelerating rotarod. EPBs were imaged for 3 days prior to training to record baseline metrics and then imaged for 3 days during forward rotarod training and again for three more days during reverse rotarod training. More than 2 weeks after the end of the rotarod training, a final imaging session was completed to capture the retention *(i.e.*, survival) of any motor training-induced structural changes. Because young animals will naturally outperform aged animals on the rotarod, unrestricted rotarod training would lead to more rotarod practice time for young animals and imbalanced opportunity for learning. To prevent this, we equalized training time across the age groups by randomly assigning each young animal to an aged animal and limiting the young animal’s training time to its aged partner’s “time to fall” on a per trial basis.

### Statistical analyses

**Fig. 1:**
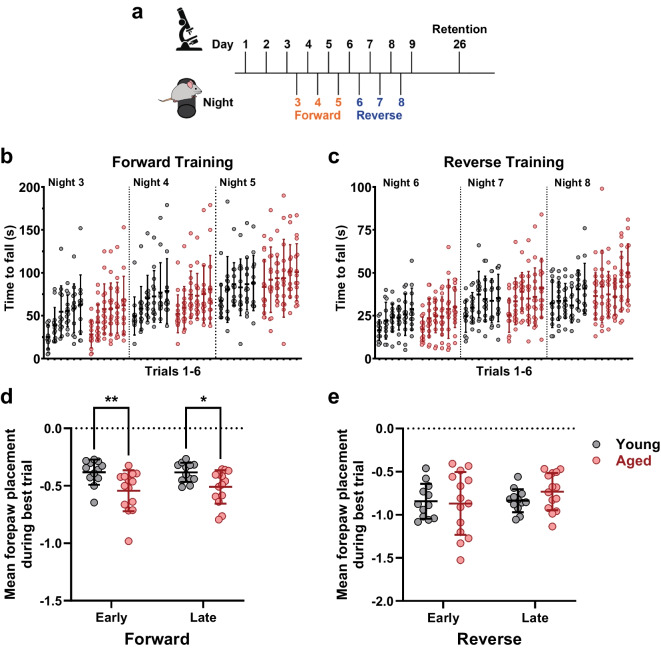
Young and aged mice undergo yoked motor skill training, and both age groups show improved performance with training. **a** Experimental protocol. Animals were imaged between 9:00 am and 2:00 pm and underwent motor training between 4:00 pm and 8:00 pm. **b** Time to fall per trial (in seconds) on each night of forward training. Black dots represent young mice; red dots represent aged mice; each column represents 1 of the 6 training trials administered per night; horizontal bar marks mean; whisker extends to ± SD. Two-way ANOVA to compare the effect of aging on performance: trial effect *p* < 0.0001, indicating trial-to-trial change; age + night effect *p* = 0.0001, indicating night-to-night change; interaction *p* = 0.8642, indicating that the effect of training was not different between age groups. **c** The same as **b** but for reverse training. Two-way ANOVA to compare the effect of aging on performance: trial effect *p* < 0.0001, indicating trial-to-trial change; age + night effect *p* < 0.0001, indicating night-to-night change; interaction *p* = 0.4447, indicating that the effect of training was not different between age groups. **d** Mean forepaw placement (in cm from top of rotarod) during the best of 6 trials on Night 3 (“Early”) and during the best of 6 trials on Night 5 (“Late”) of forward training. Two-way ANOVA to compare the effect of aging on forepaw placement: training effect *p* = 0.6237, indicating no change from early to late; age effect *p* = 0.0031, indicating an overall difference in placement between age groups; interaction *p* = 0.4447, indicating that the effect of training was not different between age groups; multiple comparisons reveal age group differences in placement during both early (*p* = 0.0082) and late training (*p* = 0.0453). **e** The same as **d** but for reverse training. Two-way ANOVA to compare the effect of aging on forepaw placement: training effect *p* = 0.2069, indicating no change from early to late; age effect *p* = 0.6288, indicating no overall difference in placement between age groups; interaction *p* = 0.2523, indicating that the effect of training was not different between age groups

**Fig. 2:**
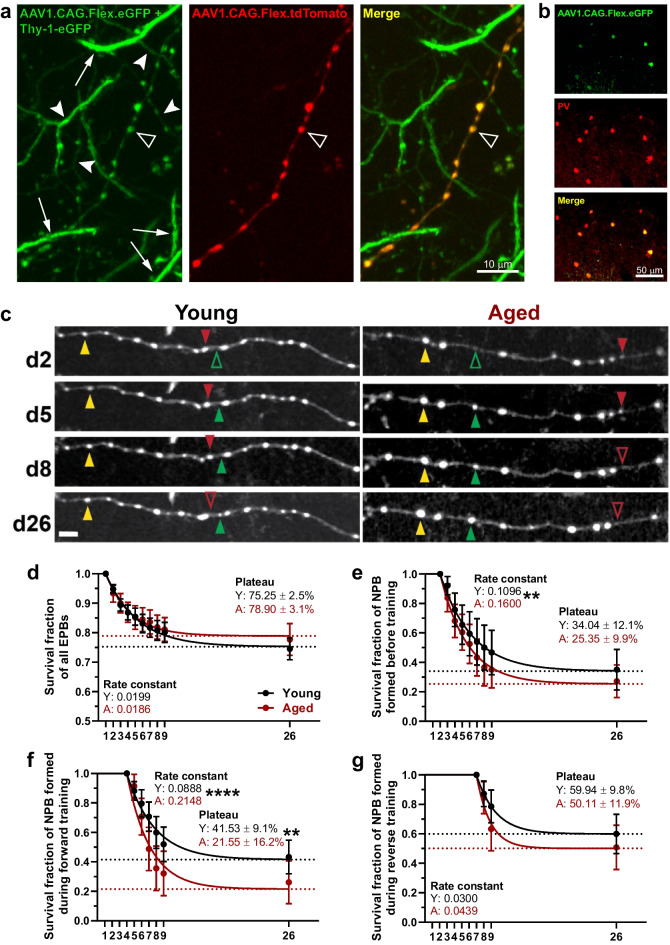
While young and aged groups display similar survival of steady-state boutons, those formed during motor training are short-lived in the aged group. **a** Labeling of PV IN axons in Thy-1-EGFP::PV-Cre mice following the injection of a mixture of AAVs (AAV1.CAG.Flex.eGFP.WPRE.bGH and AAV1.CAG.Flex.tdTomato.WPRE.bGH). Representative high-resolution images of a PV IN axon acquired with in vivo two-photon microscopy. Images are maximum intensity projections of 32 slices taken at 1.5 µm z-steps. *Left*, signal from the green channel includes pyramidal neuron dendrites (arrows), as well as of axons from excitatory neurons (arrowheads). The injection of the cre-dependent eGFP-encoding virus in these Thy-1-EGFP::PV-Cre mice added the expression of eGFP by parvalbumin interneurons (empty arrowheads); *Center*, signal from the red channel captures the tdTomato expression from the PV IN axons exclusively (empty arrowheads); *Right*, the merged signal allows the identification of axons and EPBs of PV INs. **b** Immunostaining against parvalbumin confirmed the identity of the cre-dependent fluorophore-expressing neurons as PV INs. Confocal images of putative PV INs labeled in L2/3 of Thy-1-EGFP::PV-Cre mice. *Top*, sparse eGFP expression by putative PV INs in Thy-1-EGFP::PV-Cre mice; *Middle*, staining of PV INs was done using rabbit recombinant monoclonal anti-Parvalbumin antibody (1:200, Abcam cat# ab181086, clone# EPR13091) followed by secondary goat anti-rabbit DyLight 594 (1:500, Thermo Fisher, cat# 35,560); *Bottom*, merged image demonstrating that all the eGFP-expressing cells are PV INs. **c** Representative images of young and aged axons of PV INs taken from d2 (pre-training), d5 (forward training), d8 (reverse training), and d26 (retention). Present EPBs marked with filled arrowheads; absent EPBs marked with unfilled arrowheads. Yellow, stable; green, gained; red, lost. Images are best projections of 5–10 images taken at 1.5 µm z-steps. Scale bar, 5 μm. **d** Survival of all boutons present at Day 1 of imaging. Extra sum-of-squares *F*-test to compare plateau (*p* = 0.1049) and rate constant (*p* = 0.4743). Data points represent average survival fraction per age group; solid lines, single phase exponential decay following plateau curve fit to data; dotted lines, extrapolated plateaus. Young fragments, *n* = 45; aged, *n* = 48. **e** Same as **d** but survival of all new persistent boutons formed pre-training. Extra sum-of-squares *F*-test to compare plateau (*p* = 0.2702) and rate constant (*p* = 0.0021). **f** The same as **d** but survival of all new persistent boutons formed during forward training. Extra sum-of-squares *F*-test to compare plateau (*p* = 0.0060) and rate constant (*p* < 0.0001). **g** The same as **d** but survival of all new persistent boutons formed during reverse training. Extra sum-of-squares *F*-test to compare plateau (*p* = 0.1994) and rate constant (*p* = 0.1374)

**Fig. 3:**
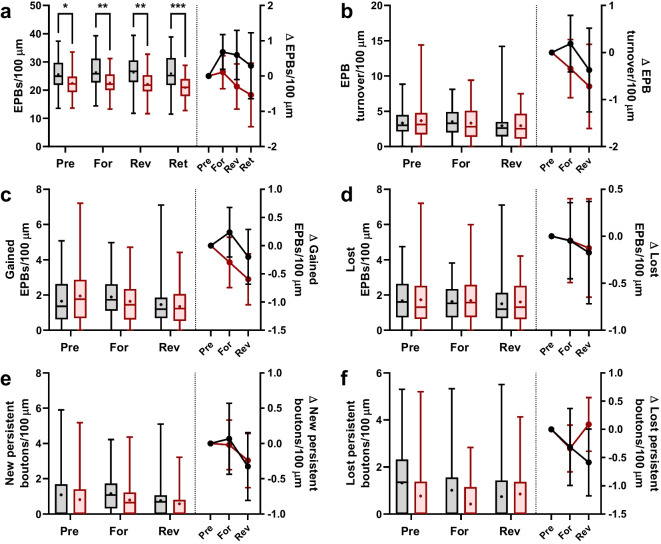
Bouton density is reduced in the aged group while bouton dynamics, which are not affected by motor training, do not differ between young and aged groups. **a** Bouton density during pre-training, forward training, reverse training, and retention stages. *Left Y-axis*, boutons per length of axon; *Right Y-axis*, change in bouton density relative to the pre-training value. Repeated measures two-way ANOVA to compare groups: training effect *p* = 0.0863, age effect *p* = 0.0001, interaction *p* = 0.0964. Multiple comparisons (by *Šidák*) between age groups: pre-pairing, *p* = 0.0227; forward, *p* = 0.0030; reverse *p* = 0.0018; retention *p* = 0.0002. **b** Bouton turnover during pre-training, forward training, and reverse training. *Left Y-axis*, bouton turnover per length of axon; *Right Y-axis*, change in bouton turnover relative to the pre-training value. **c** The same as **b**, but for boutons gained. **d** The same as **b**, but for boutons lost. **e** The same as **b**, but for gain of persistent boutons. **f**  Same as **e** but for loss of persistent boutons

**Fig. 4:**
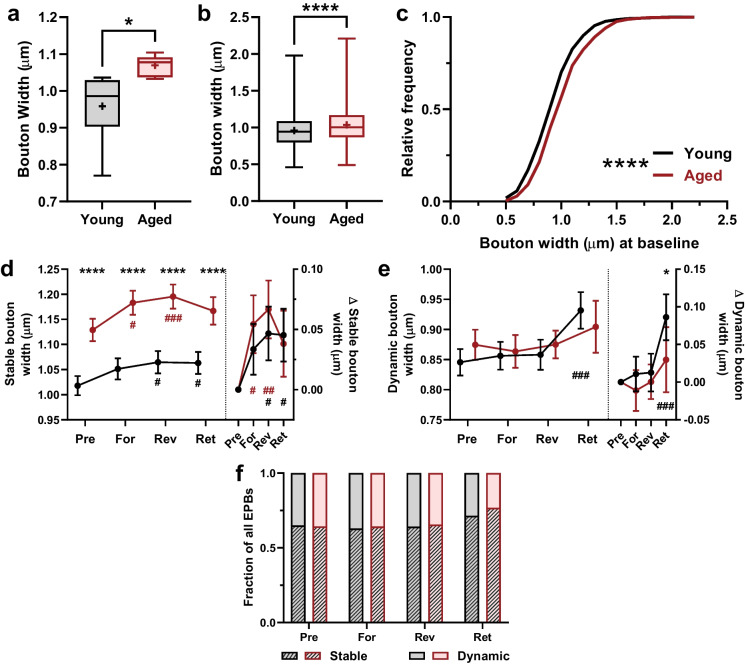
Boutons of the aged group are larger than those of the young group, driven by aging-related differences in the size of stable boutons, which is affected by motor training in both age groups. **a** Minimum-to-maximum boxplot of the average width per mouse of all boutons present during pre-pairing for each age group; horizontal line marks median, “ + ” marks mean. Young *n* = 6 mice; aged *n* = 6 mice. Unpaired *t*-test, *p* = 0.0247. **b** Minimum-to-maximum boxplot of width of all boutons present during pre-pairing for each age group; horizontal line marks median, “ + ” marks mean. Young boutons, *n* = 785; aged boutons, *n* = 844. Unpaired *t*-test, *p* < 0.0001. **c** Cumulative frequency distribution of all pre-pairing boutons. *Kolmogorov–Smirnov* test, *p* < 0.0001. **d** Width of stable boutons during pre-pairing, forward pairing, reverse pairing, and retention stages. *Left Y-axis*, bouton width in microns. Repeated measures two-way ANOVA: training effect *p* < 0.0001, age effect *p* < 0.0001, interaction *p* = 0.5252. Multiple comparisons (by *Šidák*) across age groups (marked with asterisks): pre-pairing, *p* < 0.0001; forward, *p* < 0.0001; reverse, *p* < 0.0001; retention, *p* < 0.0001. And within age groups (marked with number signs): young, pre-pairing vs. forward, *p* = 0.2799; vs. reverse, *p* = 0.0277; vs. retention, *p* = 0.0374. Aged, pre-pairing vs. forward, *p* = 0.0149; vs. reverse, *p* = 0.001; vs. retention, *p* = 0.2300; *Right Y-axis*, change in bouton width relative to pre-pairing width. Repeated measures two-way ANOVA: training effect *p* < 0.0001, age effect *p* = 0.2493, interaction *p* = 0.4219. Multiple comparisons (by *Šidák*) across age groups (marked with asterisks): forward, *p* = 0.8111; reverse, *p* = 0.8493; retention, *p* = 0.9994. And within age groups (marked with number signs): young, pre-pairing vs. forward, *p* = 0.1524; vs. reverse, *p* = 0.0080; vs. retention, *p* = 0.0119. Aged, pre-pairing vs. forward, *p* = 0.0040; vs. reverse, *p* = 0.001; vs. retention, *p* = 0.1173. **e** Width of dynamic boutons during pre-pairing, forward pairing, reverse pairing, and retention stages. *Left Y-axis*, bouton width in microns. Repeated measures two-way ANOVA: training effect *p* = 0.0002, age effect *p* = 0.5035, interaction *p* = 0.2718. Multiple comparisons (by *Šidák*) across age groups (marked with asterisks): pre-pairing, *p* = 0.6836; forward, *p* = 0.9999; reverse, *p* = 0.9875; retention, *p* = 0.9314. And within age groups (marked with number signs): young, pre-pairing vs. forward, *p* = 0.9995; vs. reverse, *p* = 0.9980; vs. retention, *p* = 0.001. Aged, pre-pairing vs. forward, *p* = 0.9997; vs. reverse, *p* = 0.9999; vs. retention, *p* = 0.8694; *Right Y-axis*, change in bouton width relative to pre-pairing width. Repeated measures two-way ANOVA: training effect *p* < 0.0001, age effect *p* = 0.0079, interaction *p* = 0.1656. Multiple comparisons (by *Šidák*) across age groups (marked with asterisks): forward, *p* = 0.8272; reverse, = 0.9973; retention, *p* = 0.041. And within age groups (marked with number signs): young, pre-pairing vs. forward, *p* = 0.9983; vs. reverse, *p* = 0.9942; vs. retention, *p* = 0.001. Aged, pre-pairing vs. forward, *p* = 0.9990; vs. reverse, *p* = 0.9998; vs. retention, *p* = 0.7507. **f** Proportion of bouton population represented by stable boutons (diagonal fill) and dynamic boutons (unfilled). *Chi-square* test to compare stable (*p* > 0.999) and dynamic (*p* = 0.1973) fractions across age groups

Statistical analyses were completed and graphs were produced using Prism (GraphPad Software, version 10.3.1 for Windows). A *p* value < 0.05 was considered statistically significant, and data are presented as the mean ± standard deviation. All statistical tests used raw data, aside from those testing differences between the change in a metric from the baseline value (for example, Fig. 3a, right). Behavioral data (Fig. 1) was compared using repeated measures two-way ANOVA (with *Geisser-Greenhouse* correction in Fig. 1b and c since the assumption of sphericity is recommended against for repeated measures) to isolate the effects of training and age. Subsequent multiple comparisons (Fig. 1d) were completed using *Šidák’s* multiple comparison test. Following fitting by least squares regression, bouton survival metrics (plateau and rate constant) were compared using the extra sum-of-squares *F*-test (Fig. 2d–g). Bouton density and turnover data (Fig. 3) and bouton width subanalysis (Fig. 4d, e) were compared using repeated measures two-way ANOVA (with *Geisser-Greenhouse* correction) and *Šidák’s* multiple comparison test. Unpaired *t*-test with *Welch’s* correction (for not assuming equal standard deviation) was used to compare bouton widths (Fig. 4b), and the *Kolmogorov–Smirnov* test was used to test for differences in the distribution of bouton widths (Fig. 4c). Stable and dynamic fractions were compared via *chi-square* tests.

## Results

### Yoked motor training allows for learning over time while legitimizing across age group comparisons

The EPBs on the axons of PV INs in the primary motor cortices of young and aged mice were imaged longitudinally over many days to monitor the density, dynamics, and morphology of these presynaptic structures. Imaging was completed in 10 sessions spread over the course of 26 days, during which the animals underwent motor training via the accelerating rotarod [37, 38], a motor learning model previously shown to induce structural plasticity in the motor cortex [6, 21–23]. After 3 days of baseline imaging, animals underwent 3 days of forward rotarod training then three more days of reverse rotarod training (Fig. 1a). A final imaging session was completed more than 2 weeks after the last rotarod training session to evaluate the retention of any motor training-induced structural changes to EPBs.

Unrestricted rotarod training would lead to imbalanced opportunity for learning between the age groups since young animals will naturally outperform their aged counterparts [35]. Therefore, we equalized training time across the age groups by randomly assigning each young animal to an aged animal and limiting the young animal’s training time to its aged partner’s “time to fall” on a per trial basis. This yoked training strategy enabled us to isolate the effect of age on this learning-related structural plasticity while still observing trial-by-trial and day-by-day performance increases over the course of forward (Fig. 1b) and reverse (Fig. 1c) motor training. While the time spent on the rotarod is effectively equalized between the age groups by yoking, it is possible that the young and aged mice use different strategies to maintain their position on the rotarod. To explore this possibility, we used post hoc analysis of training videos to track forepaw placement throughout each animal’s best trial of the day during early and late phases of motor skill training, as established previously [36]. For forward training, early and late phases were defined as Nights 3 and 5, respectively. For reverse training, they were defined as Nights 6 and 8. We found that young animal paw placement was significantly higher, *i.e.*, closer to the top of the rod, on the rotarod during both early and late phases of forward training (Fig. 1d). This indicates that the aged animals were more challenged by the forward training than the young animals since the aged animals struggled to maintain their position near the top of the rotarod. In contrast, forepaw placement during reverse training was not different between age groups (Fig. 1e), suggesting that young and aged animals were similarly challenged by the reverse training. Based on these observations, the yoked rotarod training protocol enables us to study two different styles of motor training: forward training, in which the aged group was more challenged by the task, and reverse training, in which both age groups were similarly challenged.

### Baseline boutons display similar persistence across age groups while aged boutons formed during training show reduced survival

Individual EPBs from PV INs co-expressing eGFP and tdT (Fig. 2a, b) were chronically imaged (Figs. 1a and 2c). Differences between the survival of dendritic spines present before learning and of spines gained during learning is traditionally used as an indicator that synaptic rearrangement in PNs has occurred [39, 40]. Along these lines, we examined the survival of EPBs in PV INs, beginning with the entire population of EPBs present on the first day of imaging, to look for gross differences across age groups. The rate of EPB loss and projected survival fraction revealed no aging-related differences in EPB survival over the course of the entire imaging timeline (Fig. 2d), suggesting that overall EPBs persist at similar rates in the young and aged motor cortex. Since there is evidence that motor learning evokes the formation of new EPBs on PV IN axons [19], we expected to see new, stable boutons following training. So, we narrowed our focus to only the new persistent boutons (NPBs), those gained on Day *n* then persisting at least until Day *n* + 1. We started with NPBs formed before training to look for baseline differences between the age groups. While NPBs formed before training were lost at a greater rate in the aged group, they did not differ in the ultimate survival fraction (Fig. 2e).

While aging-related differences in baseline bouton survival were slight at most, survival of boutons formed during forward training showed stark differences. The young stable fraction nearly doubled the aged stable fraction, and the aged rate of bouton elimination doubled that of the young (Fig. 2f). This indicates that the structural plasticity evoked by motor training was longer-lived in the young cortex than in the old, which is notable given that our forepaw position analysis (Fig. 1d) indicated that it is the aged animals that undergo greater challenge. This observation led us to wonder whether the short lifetime of structural plasticity was due to passive loss over time or due to active disruption brought on by the subsequent reverse training. To distinguish between these possibilities, we measured the survival of NPBs formed during reverse training and found no differences in the stable fraction or the rate of elimination (Fig. 2g). The comparable stability of NPBs between age groups after reverse training suggests that the striking difference in stability of NPBs formed during forward training may be due to aging-related deficits in presynapse stabilization, particularly in the face of new learning.

### EPB density is reduced in the aged cortex while EPB dynamics are unchanged by aging and training

The survival curves suggest that motor training is indeed promoting learning-related structural plasticity. To understand how this plasticity is deployed across the PV IN axons themselves, we next examined several metrics of structural plasticity per length of analyzed axon. First, we found that EPB density was decreased at baseline in the age group (Fig. 3a, left). This difference in EPB density persisted throughout the experimental timeline, with EPB density remaining unchanged by motor training (Fig. 3a, right). In contrast, EPB turnover per axon length did not differ between age groups (Fig. 3b, left) and was not affected by training (Fig. 3b, right). Breaking down EPB turnover into gained (Fig. 3c) and lost (Fig. 3d) components did not reveal subtler differences between age groups or effects of motor learning. Finally, the formation of NPBs (Fig. 3e) and elimination of NPBs (Fig. 3f) did not differ between age groups or reveal overt consequences of motor training. It is important to note that these dynamic EPBs make up a small fraction of all EPBs present on the axon; for example, young NPBs formed during forward training represent only 4.4% of all boutons. This is expected based on both our observed high stability of EPBs (Fig. 2d) and the scale of previous evidence of motor learning-evoked structural plasticity of PV INs [19]. This is important because it explains how the aged PV IN axon can show reduced EPB density alongside unchanged occurrence of the few dynamic EPBs but with a reduced lifetime of EPBs.

### Aging-related morphological differences between young and aged EPBs underlie differences in learning-related structural plasticity

There is evidence that corticocortical and thalamocortical axons house boutons of greater size in the aged sensory cortex than in the young [2, 8]. Based on this, we compared the width of all EPBs present on the first imaging time point (Day 1) between age groups on a per mouse basis and found that aged mice have on average larger EPBs (Fig. 4a). To capture more granular changes between age groups at the EPB population level, and as done previously by others [8, 23, 27, 41–45], we compared the width of all EPBs present during pre-pairing imaging and compared them across age groups, finding that EPBs in aged animals were indeed larger than in young on PV IN axons (Fig. 4b, c). It is possible that this increase in aged EPB size is uniformly expressed by all bouton types, or it could be that a subpopulation drives this aging-related difference. To determine between these possibilities, we divided the entire pool of EPBs into two groups based on their permanence. Each bouton was classified as either stable, if it was present throughout the entire experimental timeline, or dynamic, if it was not. The difference in bouton width was maintained in the stable group (Fig. 4d, left) but not in the dynamic group (Fig. 4e, left), indicating that the stable EPBs drive the aging-related difference in bouton size that was maintained throughout the experimental timeline (Fig. 4d, left).

Furthermore, the pool of stable EPBs also displayed motor training-related structural plasticity in the form of increased size. Stable EPBs on young axons showed a trend towards bouton enlargement during forward training that became significant during reversal training and persisted to the retention stage. Meanwhile, stable EPBs on aged axons showed an increase during forward and reverse training that was no longer detectable at the retention stage (Fig. 4d). The difference in onset is in line with the level of challenge faced by the aged groups, implied by differences in forepaw placement (Fig. 1d). Dynamic EPB width was similar between age groups across the experiment except at the retention stage, where dynamic EPBs from young mice were larger than those from aged mice (Fig. 4e). Since synapse size correlates directly with probability of persistence [8, 23], this observation dovetails with our observation of increased survival of NPBs on young axons (Fig. 2f). Taken together, these observations offer a model in which aged PV IN axons house fewer boutons per length of axon that undergo relatively transient motor training-related structural plasticity, both in the form of newly formed boutons and enlargement of existing boutons.

## Discussion

The goal of our study was to investigate whether motor training-induced differential expressions of structural plasticity in axon boutons of cortical PV INs of young and aged mice. While it is difficult to detect the potentially different effects of motor learning in the young vs. aged brain because of the expectation that the young animal can outperform the aged animal in a typical motor challenge, we addressed this potential issue with a yoked motor skill training paradigm (Fig. 1). Furthermore, our approach enabled the comparison of motor learning-induced changes in structural plasticity in two distinct training regimes: one in which the aged animals are more challenged than the young, the forward training, and another in which the young and aged animals face equal challenges, the reverse training. Our analysis of survival of new persistent boutons formed during each training regime points to the possibility of aging-related deficits in inhibitory presynapse stabilization (Fig. 2d–g). Examination of EPB density and dynamics did not uncover further effects of motor learning but did reveal, however, lower baseline EPB density on the PV IN axons of aged mice (Fig. 3). Finally, morphological analysis of individual EPBs found further baseline and motor learning-related differences between age groups. At baseline and during training, dynamic EPBs of each age group were similar in size and enlargement, while stable EPBs in the aged cortex were larger in size before and during training. However, training-related enlargement in the aged group appeared more transient, in the case of stable EPBs, and delayed, in the case of dynamic EPBs (Fig. 4). In sum, these results describe PV INs of aged mice undergoing short-lived training-evoked structural changes in the form of new EPBs with reduced survival and enlargement of existing EPBs that is less persistent than in the young cortex.

What is the likely role for PV INs during this style of motor training? The accelerating rotarod is used widely to describe motor dysfunction associated with compromised cortical PV INs [46–49] and is known to induce structural plasticity in PV INs of the primary motor cortex, M1 [24]. This is not surprising given that PV INs are known to regulate the activity of layer 2/3 and layer 5 PNs that carry cortical output [18]. Along these lines, dexterous forepaw placement is known to rely on PV IN firing prior to local PN activation and behavior onset [50]. Given that PV IN output extensively targets PN soma [51] and that PV IN activity is sharply tuned to PN activity [52], these interneurons are in prime position to modulate the activity driving skilled motor action. One of the more striking findings reported here is the aged PV IN axon’s coincident decrease in EPB density and increase in EPB size, which appeared to be driven by increased stable EPB width. This “fewer but larger” observation is consistent with other reports of high-volume presynaptic structures in the aged brain [1, 2]. Given that bouton size is a reliable indicator of synaptic strength [27, 53], it is reasonable to predict that the increase in size is an attempt to compensate for the decrease in density. Since the density of inhibitory contacts is decreased in M1 of aged mice, larger boutons may be preferentially protected (Fig. 4a and b) due to their potentially outsized influence over neighboring PNs [54]. Similar compensatory action is suggested to occur in the primary somatosensory cortex S1 [55] and dentate gyrus [56] of aged rodents.

The structural plasticity on the inhibitory PV IN axon reported here is complementary to the structural plasticity on the excitatory layer 5 PN dendrite and excitatory thalamocortical axons as well as the intrinsic excitability of the cortex, reported previously by our laboratory and others and summarized as follows: In terms of stability, we report here that EPBs on PV IN axons of young and aged mice show equal long-term stability (Fig. 2d), while the stability of spines on dendrites of L5 PNs of aged mice is reduced in M1 [3] and S1 [2, 4]. Likewise, EPBs of excitatory thalamocortical axons are also less stable in the aged brain [2, 8]. As for synaptic density, the reduced baseline EPB density reported here (Fig. 3a) is in opposition to the increased baseline dendritic spine density observed in both M1 [3] and S1 [2]. These putative structural features of aging are in line with a shift towards greater intrinsic excitability in aged S1 [16] and M1 [15]. In this regard, our laboratory has reported a decrease in the frequency of inhibitory postsynaptic currents in layer 5 PNs of aged mice, which indicates a reduction in the spontaneous release of GABA, in the number of inhibitory synapses onto PNs, or in both [16]. The human-aged brain points in a similar direction. Functional imaging shows motor control-related activity that is greater in magnitude and more widespread across brain areas than the young brain [57, 58]. An overarching hypothesis in the field is that this reflects dedifferentiation [59] of motor activity—an aging-related shift from localized, distinct activity towards diffuse activity with unclear boundaries. Recent functional imaging work in humans connects this phenomenon to reduced GABAergic inhibitory activity in the aged brain [60]. It is unclear whether this shift is maladaptive or compensatory. Negative relationships are reported between motor performance with greater contralateral [61] and ipsilateral [62] activity, as well as positive relationships between motor performance and contralateral activity [63, 64].

A few questions remain unanswered by this study. It is not clear if the structural plasticity evoked by forward training is affected by ensuing reverse training or whether this potential disruption might be more or less potent in the aged cortex. The bouton survival data may provide a hint. The NPBs formed during forward training (Fig. 2f) show greater persistence in the young cortex. Given that NPBs formed during reverse training (Fig. 2g) survive at equal rates in the young and aged cortex, one explanation is that synapse stabilization is similar between age groups when unchallenged. It may be the challenge of reversal training that compromises the survival of aged NPBs formed during forward training, suggesting that newly formed synapses along the aged axon may be more vulnerable to disruption by new learning. Forward and reversal rotarod training is known to affect spine dynamics in a similar way [22]. A related question is whether the formation of NPBs (Fig. 2f and g) or the enlargement of existing boutons (Fig. 4d) is more important for this model of motor skill learning. The only other study to follow PV IN EPBs with motor skill training (in a lever press task) found a transient increase in EPB formation that returned to baseline within a few days while changes in size were not reported [19]. While it is clear that PV INs target nearby PNs perisomatically [65], it is not yet known how these two styles of structural plasticity (formation of more boutons vs. enlargement of existing boutons) might affect the local circuit differently. Clear answers to these follow-up questions will require subsequent study using exercise and skill control groups. Aside from these newly opened questions, the results presented here highlight important qualities of the understudied aged interneuron axon at baseline and when challenged by motor training.

## Data Availability

The data generated in this study can be shared upon reasonable request.
